# A Father's Opportunity

**Published:** 1887-10-29

**Authors:** 


### A FATHER'S OPPORTUNITY.

PROBABLY the most serious wrench which the mother and her children have to undergo when death removes the head of the family, is the breaking up of the old home. To leave the house where the family has lived in happy circumstances for many years is indeed a blow not lightly to be borne. Mr. George King, the eminent actuary of the Atlas Assurance Company, 92, Cheapside, E.C., which was instituted in 1808, and has a capital of a million and a quarter, is the author of a system of mortgage assurances which enables the father of a family without difficulty to arrange for his wife to become the owner of the homestead in case of his death. The terms are so reasonable as to be surprising, and the whole scheme is one not only well worthy of the closest attention on the part of prudent men, but so munificent is it in its bearings that it ought to win the gratitude of *paterfamilias* everywhere.

